# Supplementary material for: Metabolic dysregulation contributes to the development of dysferlinopathy
Source: Life Sci Alliance. 2025 Feb 28;8(5):e202402991. doi: 10.26508/lsa.202402991 (PMC11871293; doi:10.26508/lsa.202402991)
Supplement: Supplementary file 4 [file LSA-2024-02991_TableS4.docx]

**Supplementary table**

**Table S4**. Glucose-free diet did not ameliorate dysferlinopathy.

|  | Final measures at 11 months of age | |
| --- | --- | --- |
|  | **Dysf -/- chow diet**  **(mean ± SEM)** | **Dysf -/- glucose-free diet**  **(mean ± SEM)** |
| Body weight [g] | 28.92 ± 0.467 | 26.11 ± 0.862 * |
| Fed blood glucose [mM] | 9.08 ± 0.500 | 7.26 ± 0.420 * |
| Muscle glycogen  (µg/mg muscle tissue) | 1.780 ± 0.217 | 1.779 ± 0.314 |
| Relative muscle mass [mg/g]   - Psoas - Quadriceps | 2.10 ± 0.148  6.90 ± 0.135 | 2.38 ± 0.190  6.22 ± 0.234 * |
|  |  |  |
|  | **WT chow diet (5 month)**  **(mean ±SEM)** | **WT glucose-free diet (5 month)**  **(mean ± SEM)** |
| β-hydroxybutyrate [mM] | 0.48 ± 0.062 | 0.40 ± 0.022 |

Muscle mass expressed relative to body weight. Data from 5 biological replicates. Asterisks indicate a significant difference (* *P*<0.05) compared to chow diet tested using a two-tailed Student’s t-test.
